# Comparison of 3D UTE free‐breathing lung MRI with hyperpolarized ^129^Xe MRI in pediatric cystic fibrosis

**DOI:** 10.1002/mrm.30299

**Published:** 2024-09-16

**Authors:** Samal Munidasa, Brandon Zanette, Marie‐Pier Dumas, Wallace Wee, Sharon Braganza, Daniel Li, Felix Ratjen, Giles Santyr

**Affiliations:** ^1^ Translational Medicine Program The Hospital for Sick Children Toronto Ontario Canada; ^2^ Department of Medical Biophysics University of Toronto Toronto Ontario Canada; ^3^ Division of Respiratory Medicine The Hospital for Sick Children Toronto Ontario Canada

**Keywords:** 3D free‐breathing 1‐H imaging, cystic fibrosis, hyperpolarized ^129^Xenon imaging, pulmonary ventilation, ultra‐short echo time

## Abstract

**Purpose:**

To compare phase‐resolved functional lung (PREFUL) regional ventilation derived from a free breathing 3D UTE radial MRI acquisition to hyperpolarized ^129^Xe‐MRI (Xe‐MRI), conventional 2D multi‐slice PREFUL MRI, and pulmonary function tests in pediatric cystic fibrosis (CF) lung disease.

**Methods:**

Free‐breathing 3D UTE and 2D multi‐slice ^1^H MRI as well as Xe‐MRI were acquired in 12 stable pediatric CF patients. Using PREFUL, regional ventilation (RVent) maps were calculated from the free‐breathing data. Ventilation defect percentage (VDP) was determined from 3D and 2D RVent maps (2D VDP_RVent_ and 3D VDP_RVent_, respectively) and Xe‐MRI ventilation (VDP_Xe_). VDP was calculated for the whole lung and for eight regions based on left/right, anterior/posterior, and superior/inferior divisions of the lung. Global and regional VDP was compared between the three methods using Bland–Altman analysis, linear mixed model‐based correlation, and one‐way analysis of variance and multiple comparisons tests.

**Results:**

Global 3D VDP_RVent_, VDP_Xe_, and 2D VDP_RVent_ were all strongly correlated (all *R* ^*2*^ > 0.62, *p* < 0.0001) and showed minimal, non‐significant bias (all <2%, *p* > 0.05). Three dimensional and 2D VDP_RVent_ significantly correlated to VDP_Xe_ in most of the separate lung regions (*R* ^2^ = 0.18–0.74, *p* < 0.04), but showed lower inter‐agreement. The superior/anterior lung regions showed the least agreement between all three methods (all *p* > 0.12).

**Conclusion:**

Absolute VDP assessed by 3D UTE PREFUL MRI showed good global agreement with Xe‐MRI and 2D multi‐slice PREFUL MRI in pediatric CF lung disease. Therefore, 3D UTE PREFUL MRI offers a sensitive and potentially more accessible alternative to Xe‐MRI for regional volumetric evaluation of ventilation.

## 1 INTRODUCTION

Cystic fibrosis (CF), caused by the dysfunction of the CF transmembrane regulator (CFTR) gene, is characterized by the build‐up of mucous in the airways leading to chronic lung infection and inflammation.^1^ Pulmonary MRI provides a non‐invasive, radiation‐free imaging modality for evaluating structural and functional lung abnormalities in pediatric CF^2^, ^3^ and is useful for the longitudinal monitoring of disease progression and response to CFTR modulatory therapy.^4^, ^5^ Hyperpolarized ^129^Xenon gas MRI (Xe‐MRI) provides regional pulmonary ventilation defect percent (VDP) measures and has been shown to detect early CF lung disease,^6^ capture response to antibiotic treatment for a pulmonary exacerbation,^7^ and track improvements in ventilation following therapy with the triple combination of CFTR modulators: elexacaftor, tezacaftor, and ivacaftor (ETI).^8^ However, the coached breathing maneuvers required for Xe‐MRI can be difficult to perform for young and/or very sick patients. Furthermore, the additional equipment required for Xe‐MRI (e.g., polarizer, multi‐nuclear coil, gas) and trained personnel may hinder clinical translation.

Free breathing ^1^H MRI techniques offer a contrast‐agent free methodology of assessing pulmonary ventilation and perfusion by measuring local changes in proton density following cardiorespiratory motions. These techniques include Fourier decomposition (FD) MRI,^9^ self‐gated non‐contrast‐enhanced functional lung (SENCEFUL) MRI,^10^ matrix pencil decomposition (MP) MRI,^11^ and phase‐resolved functional lung (PREFUL) MRI.^12^ Ventilation‐weighted PREFUL MRI, in particular, is performed by sorting lung MR images according to the motion of the diaphragm to interpolate high temporally resolved respiratory phases, which can be used to assess lung ventilation dynamics (e.g., flow‐volume loops).^12^, ^13^ VDP derived from 2D single‐slice PREFUL MRI has been shown to correlate well with Xe‐MRI,^14^ be a responsive measure of pulmonary exacerbation treatment,^15^ and demonstrates good repeatability in pediatric CF lung disease.^16^ Two‐dimensional multi‐slice PREFUL MRI, performed by acquiring four to five separate coronal slices of the lung, has also shown good correlation with Xe‐MRI in healthy volunteers and chronic obstructive pulmonary disease (COPD) and CF patients.^13^ However, because of the incomplete sampling of the lung with limited number of slices and/or slice gaps, ventilation defects may be missed using the 2D approach and the limited through‐plane resolution can potentially reduce detection accuracy of CF‐related pathologies perpendicular to the plane of interest, motivating the need for 3D PREFUL approaches.

More recently, a pseudo‐3D stack‐of‐stars PREFUL MRI method was developed to interrogate whole‐lung ventilation with high spatial resolution (2 mm^3^ × 2 mm^3^ × 2 mm^3^).^17^ The 3D PREFUL MRI method has been shown to correlate well with spirometry, generate repeatable PREFUL MRI ventilation maps in COPD and healthy adults,^18^ and was shown to track ventilatory improvement in CF patients at baseline and 8–16 weeks after ETI therapy.^19^ 3D approaches may benefit from the inclusion of UTE acquisitions to improve signal‐to‐noise in the lung tissue, where T_2_* is short. UTE MRI can provide high‐resolution 3D images with isotropic spatial resolution and has been used to assess pathology in CF lung disease.^20^ PREFUL with a 2D UTE sequence has been shown to be feasible in healthy adults,^21^ whereas 3D UTE‐SENCEFUL MRI has been developed using a center‐out radial trajectory, which has been shown to produce 3D ventilation maps with higher SNR as compared to the 2D approach.^21^ However, a 3D‐UTE free breathing MRI approach has not been explored in pediatric CF patients, particularly those undergoing CFTR modulator treatment, and has not been directly compared to Xe‐MRI. Although conventional PREFUL MRI and Xe‐MRI have been compared in pediatric CF lung disease, previous PREFUL MRI analysis was limited to a single slice. We hypothesize that the proposed 3D UTE PREFUL MRI technique will provide a whole lung ventilation assessment, which more closely agrees with multi‐slice Xe‐MRI.

The objective of this study was to determine the feasibility of obtaining regional ventilation maps and corresponding VDP using 3D UTE PREFUL in pediatric CF lung disease and compare the method to multi‐slice Xe‐MRI and PREFUL MRI, as well as pulmonary function tests (PFTs).

## 2 METHODS

### 2.1 Subjects and pulmonary function tests

Twelve stable pediatric CF patients performed pulmonary function tests (spirometry and nitrogen gas multiple‐breath washout [MBW]) and underwent same‐day imaging using a clinical 3 T scanner (MAGNETOM Prismafit, Siemens Healthcare). Five participants were imaged 3 to 12 months after their first visit. The participants were recruited through a protocol approved by The Hospital for Sick Children (REB 1000063021; Clinical‐Trials.gov NCT04391322) and Health Canada as part of an ongoing, multi‐site study assessing the response of Xe‐MRI and pulmonary function changes to CFTR‐modulator treatment in pediatric CF patients (HyPOINT‐Part 2: REB 1000077493; Clinical‐Trials.gov NCT04259970). Written informed assent/consent was obtained from the legal guardian or parent of each participant before examination.

Spirometry^22^ (V_max_, VIASYS CareFusion) as well as N_2_ MBW^23^ (Exhalyzer D, EcoMedics AG) were performed by trained professionals according to American Thoracic Society guidelines. The percent predicted forced expiratory volume in 1 s (FEV_1_% predicted) and the lung clearance index (LCI) were determined from spirometry and N_2_ MBW, respectively.

### 2.2 MRI

#### 2.2.1 Xe‐MRI

HP ^129^Xe images were acquired with a flexible ^129^Xe vest coil (Clinical MR Solutions), using isotopically enriched ^129^Xe (85%) polarized to ˜25% (Model 9820, Polarean). The HP ^129^Xe dose was set to 1/10th of the participant's total lung capacity (TLC) (estimated using patient height and sex) and balanced with medical‐grade N_2_ to a total volume of 1/6th of the participant's TLC. The participant was coached to inhale the dose bag from functional residual capacity (FRC) and, using a fast spoiled gradient recalled echo (GRE) sequence, 10 to 14 coronal slices were acquired during a 6‐ to 8‐s breath‐hold. As previously described,^16^ the following scan parameters were used: TR/TE = 7.8 ms/2.78 ms, flip angle = 10°, FOV = 360 × 384 mm^2^, matrix = 180 × 192, slice thickness = 15 mm, and bandwidth (BW) = 170 Hz/pixel, 62.5% with partial echo and centrically ordered phase ordering. Volume‐matched anatomical ^1^H images were acquired with a 3D‐FLASH sequence using a combination of a torso array and spine coil (Siemens Healthcare) as previously reported.^16^

Following Xe‐MRI acquisition, a N4ITK bias‐field correction was applied to the images in 3D Slicer (http://www.slicer.org)^24^ and was registered to the corresponding ^1^H images. Using MATLAB, a thoracic cavity mask was generated from anatomical ^1^H MR images using a 2D U‐net based deep learning segmentation approach.^25^ A signal intensity threshold of 60% of the mean signal was used to determine the defect region within the masked ^129^Xe images and VDP was determined as the total defect volume as a percentage of the total lung volume.^26^

#### 2.2.2 Free breathing MRI

**FIGURE 1:**
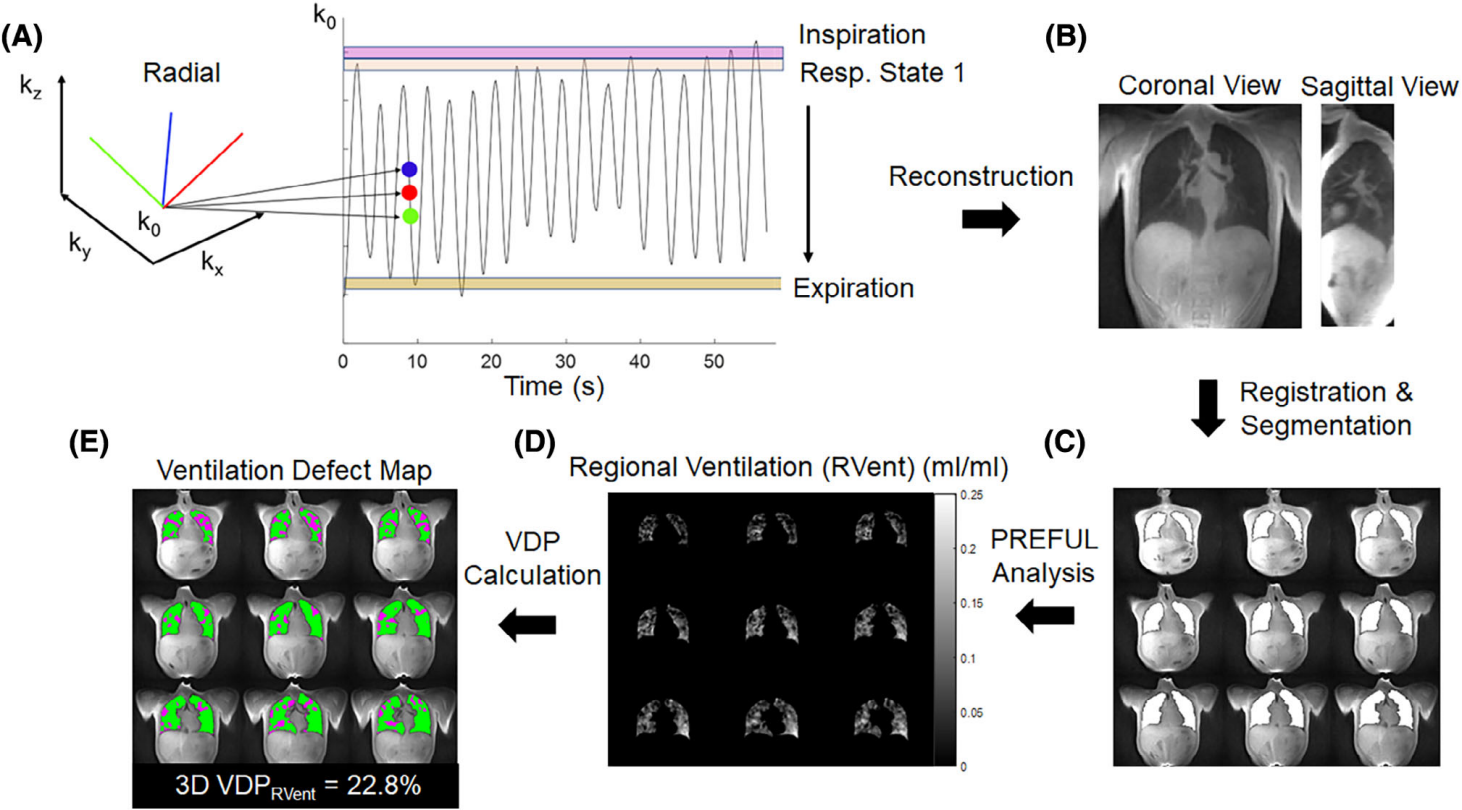
MATLAB‐based post processing pipeline for the 3D UTE phase‐resolved functional lung (PREFUL) MRI analysis. In brief, (A) the direct current (DC) signal of a single coil element closest to the diaphragm was used to sort the radial spokes into 30 respiratory phases. (B) Each respiratory phase was reconstructed with ˜21 000 spokes. (C) Each image was registered to the mid‐respiration state, spatially fitlering was applied, and the thoracic cavity was segmented . (D) Following the PREFUL algorithm, 15 equidistant phases were interpolated using non‐parametric regression. The regional ventilation (RVent) of each voxel within the thoracic cavity mask was determined and (E) a corresponding ventilation defect percentage (VDP) map was calculated using k‐means clustering.

The 3D free breathing acquisition was performed using a 3D UTE sequence with a 20 μs non‐selective hard pulse and a center‐out 3D golden‐means radial “koosh‐ball” trajectory^27^ with ramped sampling. As described above, ^1^H MRI images were acquired using a torso array and spine coil for a total of 30 receiving coil elements. The acquisition was performed with the following scan parameters: TR/TE = 1.92 ms/ 0.05 ms, FOV = 500 × 500 × 500 mm^3^, BW = 830 Hz/pixel, and flip angle = 3°. A total of 280 000 radial spokes were acquired for a total acquisition time of 8 min and 58 s. Figure 1 summarizes the post‐processing pipeline for the 3D UTE PREFUL MRI analysis. For each participant, the average signal magnitude of the first five readout points (i.e., direct current [DC], signal) from a single coil element closest to the diaphragm was used to determine a respiratory waveform. The respiratory waveform was temporally filtered using a Savitzky–Golay function.^21^ To address the drift in the waveform's amplitude, the DC signal was rescaled from 0 to 1 using the minimum and maximum values within consecutive 5000‐spoke increments. A binning window was run across the DC signal to reconstruct 30 respiratory phases from expiration to inspiration using ˜21 000 spokes per state. Approximately 30% of the spokes were shared between neighboring states to increase apparent SNR by reducing under sampling artifacts. The weighting of these shared spokes at the periphery of the binning window was determined using a decaying exponential function as described by Jiang et al.^28^

Each respiratory phase was reconstructed to a resolution of 2 × 2 × 2 mm^3^ by applying iterative SENSE with the gpuNUFTT toolbox in MATLAB.^29^ Here, coil sensitivity maps were estimated from non‐SENSE reconstructed 3D images by applying adaptive coil combination. Regularization was applied along the spatial dimension using 3D total variation (λ_1_ = 0.01) and 3D Symlet 4 wavelet (λ_2_ = 0.01) regularization terms. Each image was registered to the mid‐respiratory phase using a polynomial non‐rigid registration using the Forsberg Registration Toolbox.^30^ The thoracic cavity was segmented using a 3D seeded region‐growing algorithm and a 3D image‐guided filter was applied. Following the PREFUL algorithm,^12^, ^17^ 15 equidistant phases were interpolated using non‐parametric regression and the regional ventilation (RVent) of each voxel within the thoracic cavity mask was determined. As a comparator, 2D PREFUL analysis was performed on five separately acquired 2D coronal slices. The first slice was positioned slightly posterior to the heart, one slice was posterior to the first slice, whereas the remaining three slices were spaced equidistantly toward the anterior direction. The following acquisition parameters were used: TR/TE = 0.99 ms/0.73 ms, flip angle = 5°, FOV = 500 × 500 mm^2^, matrix = 128 × 128 interpolated to 256 × 256, slice thickness = 15 mm, BW = 1500 Hz/pixel, phase partial Fourier = 6/8. A total of 512 repetitions of the slice were acquired in ˜61 s using a TurboFLASH sequence for a total average duration of ˜5 min and PREFUL analysis was performed in MATLAB (R2021a, The MathWorks) as previously described.^16^ For both the 2D and 3D UTE PREFUL MRI analysis, RVent was calculated within the thoracic cavity mask pixel‐wise according to Klimes et al.^17^ Two dimensional and 3D k‐means clustering approaches^16^, ^31^ were used to determined 2D and 3D VDP_RVent_, respectively.

### 2.3 Statistical analysis

All statistical analyses were performed using MATLAB. Patient demographics, 3D VDP_RVent_, 2D VDP_RVent_, VDP_Xe_, FEV_1_ (% pred.), forced vital capacity (FVC) (% pred.), FEV_1_/FVC (% pred.), and LCI data were reported as a median and range. Bland–Altman analysis was used to assess agreement between VDP metrics determined by Xe‐MRI and 2D or 3D UTE PREFUL MRI and significant differences, between and within subjects, were compared using a one‐way analysis of variance with a multiple comparison test using a Bonferroni correction. A linear mixed‐effects model was used to correlate the VDP between three MRI methods using the R statistical software^32^ to separate the fixed effects from the random effects (i.e., the within‐subject correlation). A marginal coefficient of determination, *R* ^*2*^, was determined to measure the correlation strength solely because of the fixed effects and corresponding *p*‐values were determined using Satterthwaite approximation.^33^, ^34^ Spatial overlap of VDP_Xe_ with 2D or 3D UTE PREFUL MRI‐derived VDP_RVent_ was evaluated using a Dice similarity coefficient (DSC) to determine spatial agreement of the healthy ventilatory and defect regions detected by the two imaging modalities. The 3D UTE PREFUL MRI defect maps were averaged in the anterior–posterior direction to match the out‐of‐plane resolution of the Xe‐MRI defect maps and a landmark‐based method was used to register slice‐matched defect maps before DSC calculation. For comparisons between 2D PREFUL MRI and Xe‐MRI, slices were visually matched and registered to compute DSC values.

In addition to the calculation of DSC, a regional comparison of ventilation defects was investigated by determining VDP separately at eight different regions of the lung. First, the left and right lungs were separated. To separate the superior (upper) and inferior (lower) region of the lung, the axial position approximating the position of the carina was used as the separation point. Similarly, the sagittal position best approximating the position of the carina was used to separate the anterior and posterior lung. Although the position of the carina was discerned directly from the corresponding ^1^H MR images used to generate the 3D UTE and 2D multi‐slice PREFUL MRI regional ventilation maps, the anatomically matched ^1^H MR images obtained for Xe‐MRI analysis were used to divide the Xe‐MRI ventilation maps. Correlation and significant differences between regional VDP of the three methods were determined similar to global VDP as described above.

A *p*‐value <0.05 was considered statistically significant for all tests.

## 3 RESULTS

**FIGURE 2:**
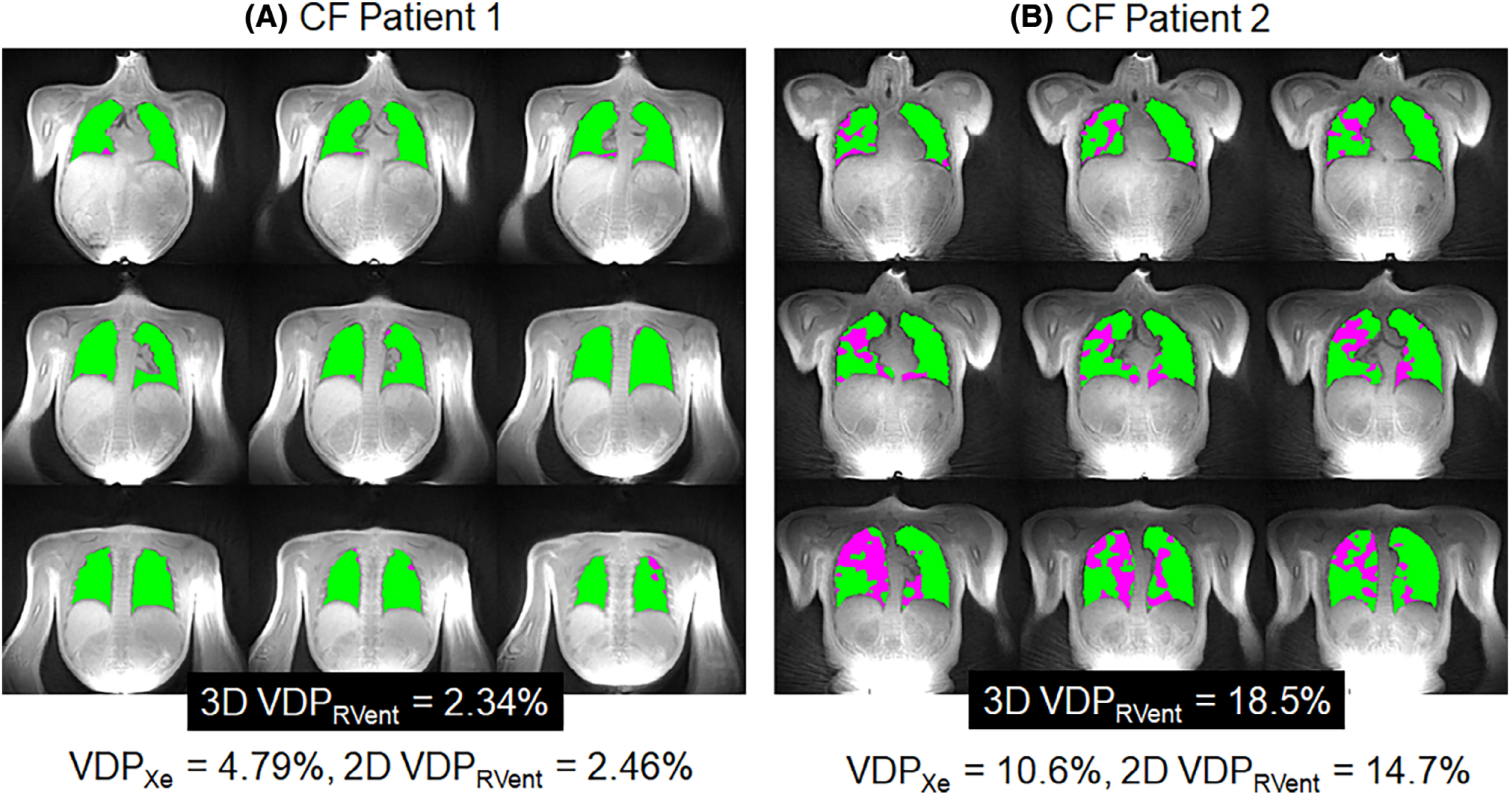
Ventilation defect maps derived from 3D UTE phase‐resolved functional lung (PREFUL) MRI regional ventilation (RVent) distributions for two representative pediatric CF patients with (A) low 3D RVent VDP (3D VDP_RVent_) and (B) high 3D VDP_RVent_. Hy ^129^Xe‐MRI derived VDP (VDP_Xe_) and 2D PREFUL MRI RVent VDP (2D VDP_RVent_) are shown for each CF patient. CF, cystic fibrosis; VDP, ventilation defect percentage.

**FIGURE 3:**
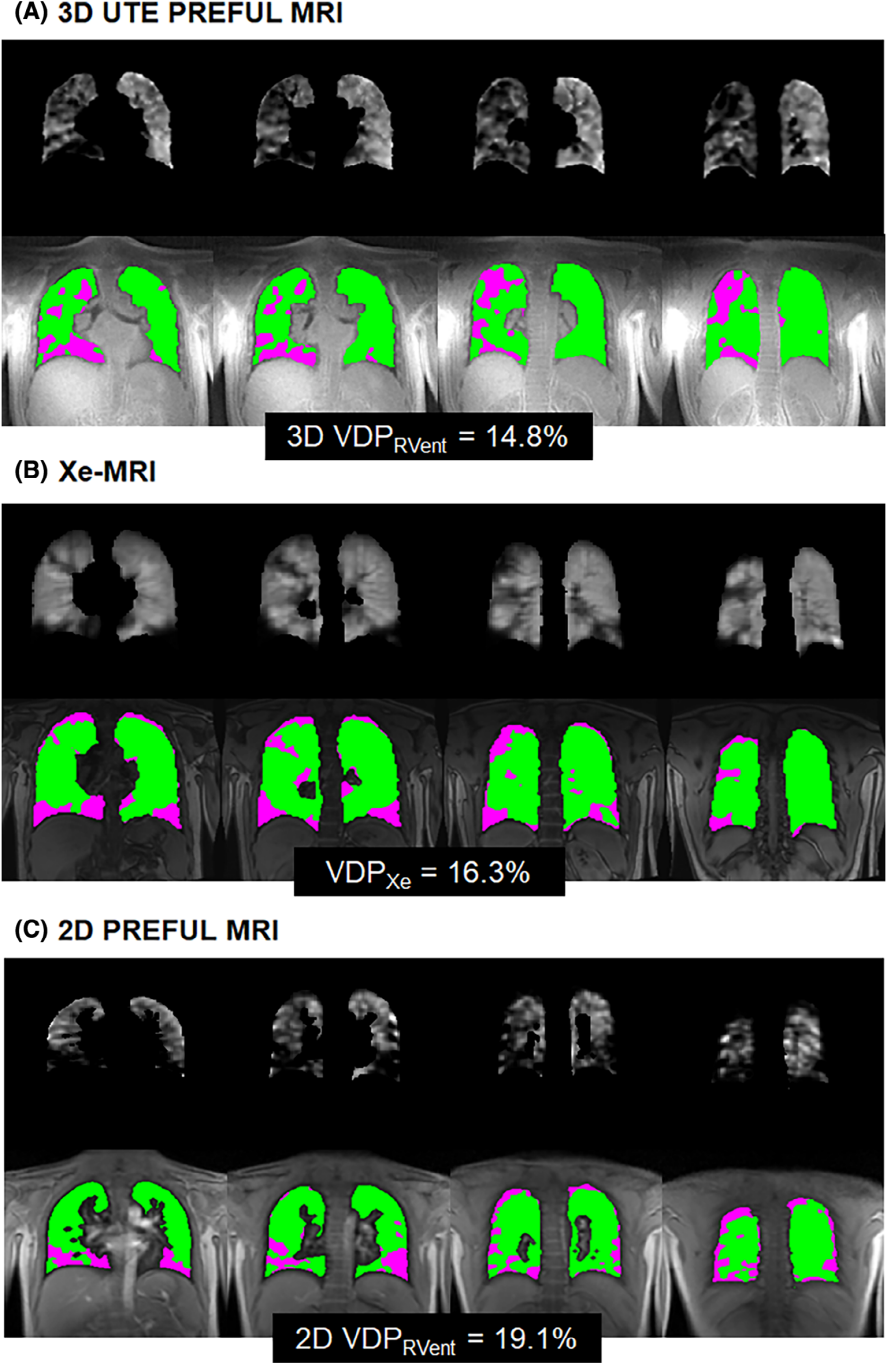
Normalized and segmented, gray‐scaled ventilation maps and corresponding defect maps derived from (A) 3D UTE phase‐resolved functional lung (PREFUL) MRI (B) ^129^Xe‐MRI (Xe‐MRI) (C) and 2D PREFUL MRI for a representative pediatric CF patient. Similar ventilation defects can be found at the inferior region of both lungs and at the superior region of the right lung toward the posterior slices. Dice coefficients for healthy and defect regions, respectively, are: 0.75 and 0.24 for 3D VDP_RVent_ and VDP_Xe_, 0.77 and 0.16 for 3D VDP_RVent_ and 2D VDP_RVent_, and 0.75 and 0.21 for 2D VDP_RVent_ and VDP_Xe_. CF, cystic fibrosis; VDP, ventilation defect percentage.

**FIGURE 4:**
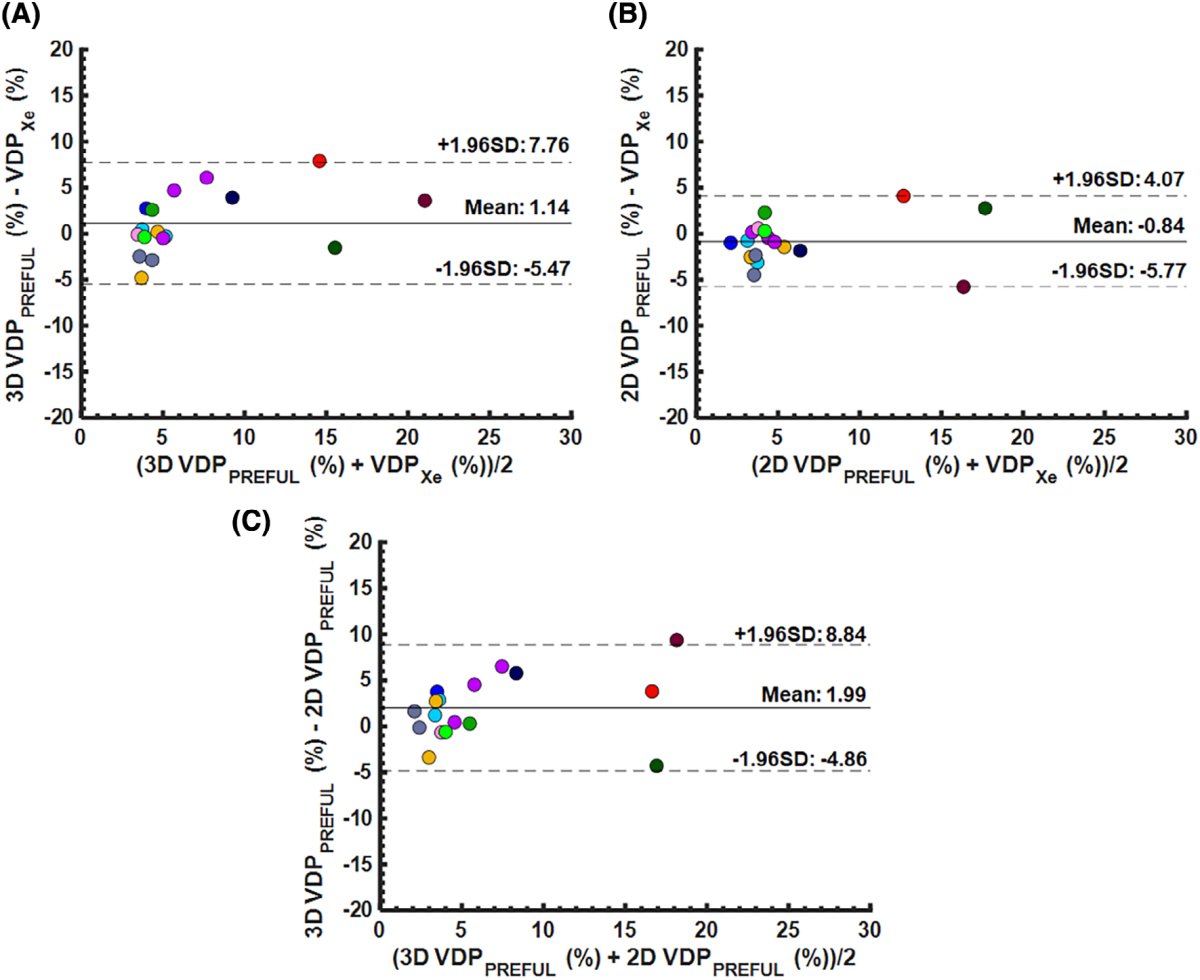
Bland–Altman analysis of (A) 3D UTE phase‐resolved functional lung (PREFUL) MRI regional ventilation (RVent) VDP (3D VDP_RVent_) and ^129^Xe‐MRI (Xe‐MRI) VDP (VDP_Xe_), (B) 2D PREFUL MRI RVent VDP (2D VDP_RVent_) and VDP_Xe_, and (C) 3D VDP_RVent_ and 2D VDP_Rvent_ in pediatric CF patients. Each subject is represented by a unique color. The solid black line and dashed black lines represent the mean bias and the 95% confidence interval, respectively. A mean bias of 1.14% (−5.47, 7.76) was found between 3D VDP_RVent_ and VDP_Xe_, and − 0.84% (−5.77, 4.07) between 2D VDP_RVent_ and VDP_Xe_. The mean bias between 3D VDP_RVent_ and 2D VDP_RVent_ was 1.99% (−4.86, 8.84). CF, cystic fibrosis; VDP, ventilation defect percentage.

**FIGURE 5:**
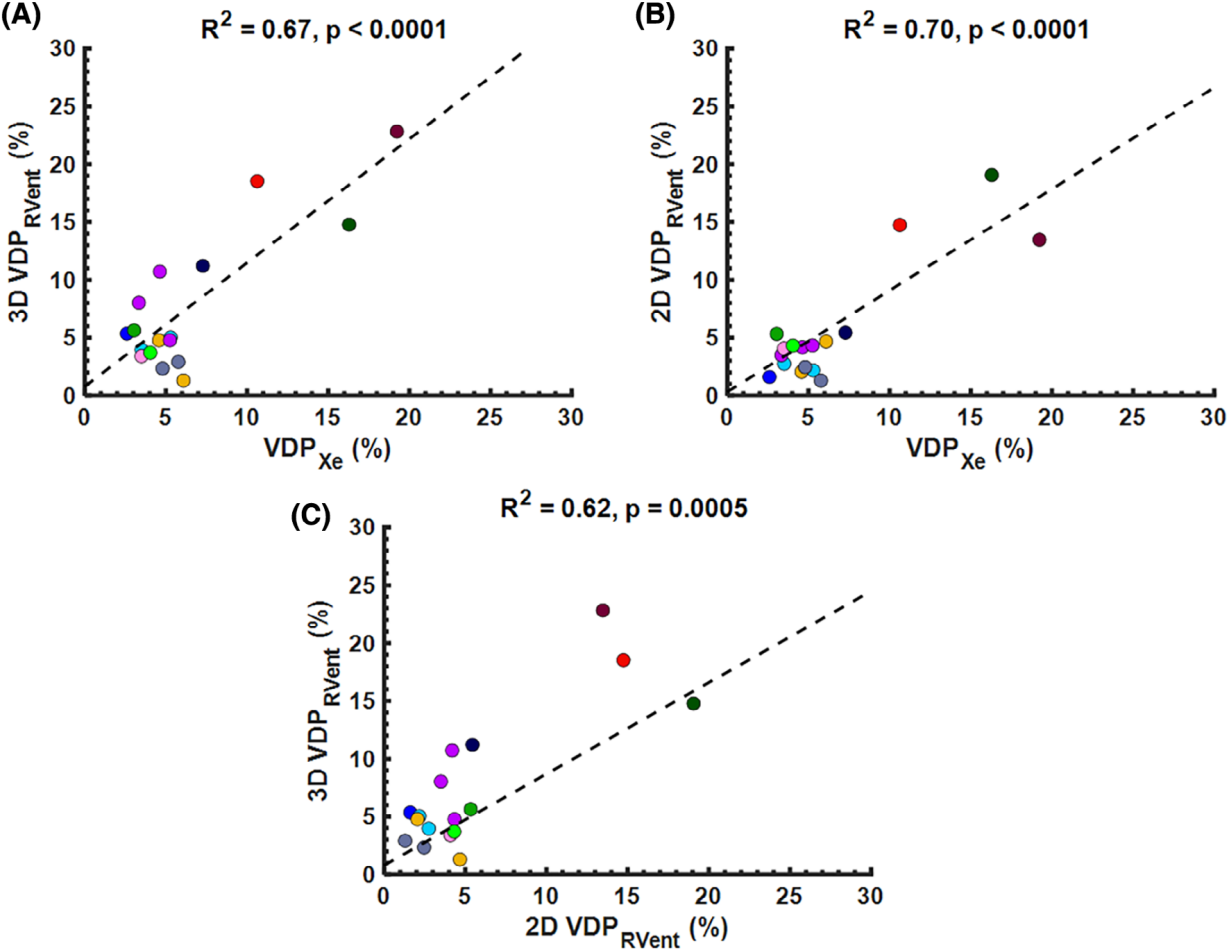
Correlation plots between (A) 3D UTE phase‐resolved functional lung (PREFUL) MRI regional ventilation (RVent) VDP (3D VDP_RVent_) and ^129^Xe‐MRI (Xe‐MRI) VDP (VDP_Xe_), (B) 2D PREFUL MRI RVent VDP (2D VDP_RVent_) and VDP_Xe_, and (C) 3D VDP_RVent_ and 2D VDP_RVent_ in pediatric CF patients. Each subject is represented by a unique color. Significant correlations are considered to be *p* < 0.05. CF, cystic fibrosis; VDP, ventilation defect percentage.

MRI and pulmonary function tests were feasible for all CF participants (*n* = 12; 7 male: 5 female). Table 1 summarizes the patient demographics, pulmonary function measures (FEV_1_ and LCI), and MRI outcomes for the pediatric CF patients. None of the VDP metrics within the cohort of CF patients were significantly different from each other (*p* > 0.05). Figure 2 shows the ventilation defect maps derived from 3D UTE PREFUL MRI from two representative CF patients. The CF patient with a 3D VDP_RVent_ of 2.34% showed correspondingly low VDP_Xe_ (4.79%). The CF patient with a 3D VDP_RVent_ of 14.8% (where ventilation defects are situated in the right lung) showed high VDP_Xe_ (16.3%). Figure 3 shows the ventilation defect maps derived from 3D UTE PREFUL MRI, Xe‐MRI, and 2D PREFUL MRI from a representative CF patient in which similar disease severity and regional ventilation defects can be observed using all three methods. Bland–Altman analysis of the VDP_Xe_, 2D VDP_RVent_, and 3D VDP_RVent_ are shown in Figure 4. Correlation plots between VDP_Xe_ and 2D and 3D VDP_RVent_ across all three time points are shown in Figure 5. All correlations were statistically significant (all *R* ^2^ > 0.62, *p* < 0.0005). LCI significantly correlated with 3D VDP_RVent_ (*R* ^2^ = 0.14, *p* = 0.03) and VDP_Xe_ (*R* ^2^ = 0.30, *p* = 0.02), but not 2D VDP_RVent_ (*p* = 0.20). 3D VDP_RVent_, 2D VDP_RVent_, and VDP_Xe_ did not significantly correlate with FEV_1_, FVC, or FEV_1_/FVC (all *R* ^2^ < 0.06 and *p* > 0.08).

**TABLE 1:** Summary of participant demographics, pulmonary function measures, and Xe‐MRI and PREFUL MRI‐derived parameters in all pediatric cystic fibrosis patients.

Characteristic	Value
No. of participants	12
Total no. of visits	17
Age	16 (12–19)
FEV_1_ (% predicted)	93 (66–120)
FVC (% predicted)	100 (74–121)
FEV_1_/FVC (% predicted)	94 (77–100)
LCI	6.4 (4.6–9.2)
VDP_Xe_ (%)	4.8 (2.6–19)
3D VDP_RVent_ (%)	5 (1.3–23)
2D VDP_RVent_ (%)	4.2 (1.3–19)

*Note*: Values are reported as median (range).

Abbreviations: FEV_1_, forced expiratory volume in 1 s; FVC, forced vital capacity; LCI, lung clearance index; PREFUL, phase‐resolved functional lung; RVent, regional ventilation; VDP, ventilation defect percentage; Xe, hyperpolarized ^129^Xe‐MRI.

**FIGURE 6:**
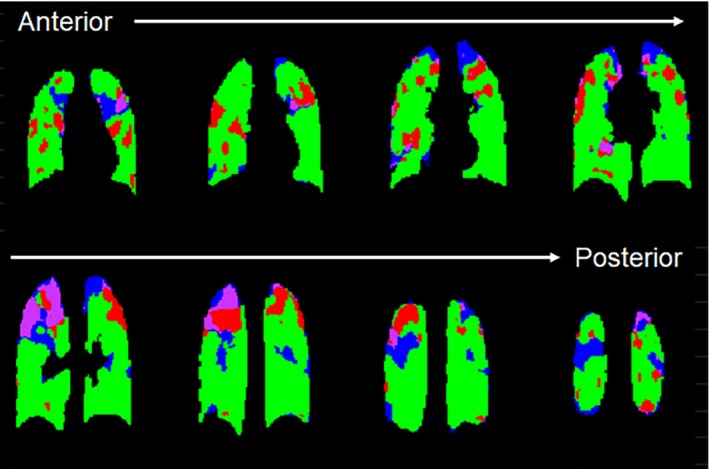
The overlap of the ^129^Xe‐MRI (Xe‐MRI) and 3D phase‐resolved functional lung (PREFUL) MRI derived ventilation defects are shown for all lung slices (anterior/posterior) in a representative pediatric CF patient, with 3D VDP_RVent_ = 22.9% and VDP_Xe_ = 19.2% (purple regions = both 3D VDP_RVent_ and VDP_Xe_ red regions = only 3D VDP_RVent_ blue regions = only VDP_Xe_ and green regions = neither VDP_Xe_ nor 3D VDP_RVent_). The Dice similarity coefficient (DSC) values of the healthy and defect regions are 0.75 and 0.24, respectively. CF, cystic fibrosis; VDP, ventilation defect percentage.

Figure 6 shows the overlapping ventilation defect maps derived from both Xe‐MRI and 3D UTE PREFUL MRI in a representative subject. Moderate spatial correspondence of the VDP_Xe_ and 3D VDP_RVent_ is shown in this patient. Overall, the median DSC values of the healthy ventilatory regions of all three methods were within 0.66 to 0.80, whereas the median DSC values of the defect regions were low, ranging from 0 to 0.05, where the highest DSC was 0.29. No pair of methods showed significantly better spatial overlap (*p* > 0.05). The correlations between 3D VDP_RVent_, VDP_Xe_, and VDP_RVent_ determined at specific regions of the lung are shown in Table 2, and the median VDP at these regions are shown in Table 3. Overall, a majority of the regional VDP values were strongly correlated between the three methods, where the least correspondence was found at the superior, anterior region of both the left and right lungs. Notably, 3D VDP_RVent_ was significantly lower at the superior, anterior region of the right lung, whereas 2D VDP_RVent_ was significantly lower in the same regions of both the right and left lung.

**TABLE 2:** Correlation of 3D VDP_RVent_, 2D VDP_RVent_, and VDP_Xe_ per region of the lung.

Lung region	3D VDP_RVent_ vs. VDP_Xe_	2D VDP_RVent_ vs. VDP_Xe_	3D VDP_RVent_ vs. 2D VDP_RVent_
Right/superior/anterior	*R* ^*2*^ = 0.05, *p* = 0.32	*R* ^*2*^ = 0.14, *p* = 0.12	*R* ^*2*^ = 0.02, *p* = 0.44
Left/superior/anterior	*R* ^*2*^ = 0, *p* = 0.94	*R* ^*2*^ = 0, *p* = 0.88	*R* ^*2*^ = 0.14, *p* = 0.08
Right/inferior/anterior	***R*** ^***2***^ = **0.66, *p* < 0.0001**	***R*** ^***2***^ = **0.42, *p*** = **0.004**	*R* ^*2*^ = 0.01, *p* = 0.43
Left/inferior/anterior	***R*** ^***2***^ = **0.23, *p*** = **0.04**	***R*** ^***2***^ = **0.74, *p* < 0.0001**	*R* ^*2*^ = 0.02, *p* = 0.40
Right/superior/posterior	***R*** ^***2***^ = **0.47, *p*** = **0.003**	***R*** ^***2***^ = **0.40, *p*** = **0.007**	***R*** ^***2***^ = **0.43, *p*** = **0.006**
Left/superior/posterior	***R*** ^***2***^ = **0.34, *p*** = **0.01**	***R*** ^***2***^ = **0.28, *p*** = **0.02**	***R*** ^***2***^ = **0.27, *p*** = **0.03**
Right/inferior/posterior	***R*** ^***2***^ = **0.18, p** = **0.02**	***R*** ^***2***^ = **0.57, *p*** = **0.0009**	***R*** ^***2***^ = **0.58, *p*** = **0.0004**
Left/inferior/posterior	*R* ^*2*^ = 0.12, *p* = 0.16	*R* ^*2*^ = **0.65, *p*** = **0.0001**	*R* ^*2*^ = 0.01, *p* = 0.78

*Note*: Significant correlations (*p* < 0.05) are bold.

Abbreviations: RVent, regional ventilation; VDP, ventilation defect percentage; Xe, ^129^Xe‐MRI.

**TABLE 3:** 3D VDP_RVent_, 2D VDP_RVent_, and VDP_Xe_ per region of the lung.

Lung region	3D VDP_RVent_ (%)	VDP_Xe_ (%)	2D VDP_RVent_ (%)	3D VDP_RVent_ vs. VDP_Xe_ (*p*‐value)	2D VDP_RVent_ vs. VDP_Xe_ (*p*‐value)	3D VDP_RVent_ vs. 2D VDP_RVent_ (*p*‐value)
Right/superior/anterior	2.3 (0–22)	10 (2.9–28)	0.98 (0–15)	**0.017**	**0.0003**	0.52
Left/superior/anterior	2.4 (0–27)	10 (0.9–29)	1 (0–6.4)	0.63	**0.0027**	0.083
Right/inferior/anterior	7.5 (0.84–30)	3.8 (1.3–12)	2.8 (0–38)	0.23	0.97	1
Left/inferior/anterior	3.1 (0.42–39)	2.7 (0.39–14)	2.1 (0–30)	1	1	1
Right/superior/posterior	5.7 (0.11–54)	8.8 (2–57)	10 (1.8–31)	1	1	1
Left/superior/posterior	4.2 (0.2–32)	5.7 (1.3–31)	6.2 (1.4–17)	1	1	1
Right/inferior/posterior	4.8 (1.2–32)	3 (0.33–23)	2.3 (0–26)	0.37	1	0.24
Left/inferior/posterior	3.5 (0.74–23)	3.7 (0.55–13)	4.4 (0.56–19)	1	1	1

*Note*: Values are reported as median (range). Significant differences (*p* < 0.05) are indicated in bold.

Abbreviation: RVent, regional ventilation; VDP, ventilation defect percentage; Xe, ^129^Xe‐MRI.

## 4 DISCUSSION

This study demonstrates the feasibility of applying the PREFUL method with a 3D golden‐means UTE MR acquisition in pediatric CF patients. Three dimensional PREFUL MRI captures regional ventilation with a higher spatial resolution (2 × 2 × 2 mm^3^) and lung coverage as compared to 2D PREFUL MRI (2 × 2 × 15 mm^3^) for a marginal increase in scan time. The main finding of the study was global VDP derived from the proposed 3D UTE PREFUL MRI method (3D VDP_RVent_) correlated strongly with multi‐slice Xe‐MRI derived VDP (VDP_Xe_) similar to the conventional 2D multi‐slice PREFUL method, whereas regional VDP showed some instances of discordance.

In the pediatric CF participants, 3D VDP_RVent_ showed a comparable absolute bias and correlation strength to VDP_Xe_ as compared to 2D multi‐slice VDP_RVent_. Here, the strong correlation between 2D multi‐slice VDP_RVent_ and VDP_Xe_ was comparable to a previous study in which 2D single‐slice VDP_RVent_ correlated to VDP_Xe_ in a different cohort of pediatric CF patients.^16^ In contrast to the work of Couch et al.,^14^ which showed a mean bias of 9.0% ± 11.5% between single‐slice PREFUL VDP and Xe‐MRI VDP in a cohort of stable pediatric CF patients and CF patients undergoing a pulmonary exacerbation (PEx), the bias between 3D and 2D multi‐slice PREFUL VDP were 0.84% and 1.14%, respectively. Although the reduction in bias between the two techniques may be attributed to the inclusion of multiple slices when performing PREFUL MRI, the differences in VDP quantification may also be a contributing factor to this discrepancy. Specifically, Couch et al.^14^ used k‐means clustering to determine VDP for both Xe‐MRI and PREFUL MRI ventilation distributions. Whereas, in the present study, a threshold of 60% of the mean signal intensity was used to determine VDP from Xe‐MRI, although k‐means clustering was used to quantify ventilation defects in PREFUL MRI. In a previous study comparing 2D multi‐slice PREFUL MRI to Xe‐MRI in adult COPD and CF patients, and healthy volunteers, the VDP of the two methods was found to significantly correlate and moderate spatial overlap was found.^13^ In that study, a linear binning method (which uses a healthy cohort's average ventilation distribution as reference) as well as a hard threshold of 0.4 × 90th percentile of the ventilation signal were used to quantify ventilation defects for both Xe‐MRI and PREFUL MRI and, in both cases, no significant biases were found. Although the absolute quantification of VDP derived from the three MRI methods correlated strongly, the median regional overlap of ventilation defects, as measured by the DSC, was low. The low spatial overlap of defect regions can likely be attributed to a combination of the fundamental differences of the MRI methods and the misregistration of small defect volumes. The findings are consistent with the low DSC values (<0.05) of defect regions found from PREFUL MRI and Xe‐MRI previously reported in healthy and stable CF patients, and moderate overlap (˜0.20) in CF patients undergoing a PEx.^14^ In comparison, Kaireit et al.^13^ reported median DSC values between 2D multi‐slice PREFUL MRI derived VDP_RVent_ and VDP_Xe_ of ˜0.34 in a cohort of CF and mostly COPD patients, where disease severity was higher. Nevertheless, spatial agreement of healthy regions was much higher in agreement with both these past studies.^13^, ^14^

To circumvent the difficulty in aligning ventilation defects between the three methods, a regional comparison of VDP was performed by anatomically separating the lung into eight regions based on the right/left, superior/inferior, and anterior/posterior positions. Using this analysis method, 3D VDP_RVent_, and 2D VDP_RVent_ showed strong correlation to VDP_Xe_ in a majority of the separate lung regions. However, there were less regions showing significant correlation between 3D and 2D VDP_RVent_, which could be attributed to the larger discrepancy in the acquired spatial resolution and total lung volume of the two methods as well as differences in their estimation of ventilation. For instance, the 3D PREFUL method captures changes in lung density in both the in‐plane and through‐plane direction whereas the 2D method only captures the former. Additionally, the determination of the respiratory phases using the 2D PREFUL is easily captured by tracking the motion of the diaphragm across coronal slices, whereas reconstruction of respiratory phases from the DC signal is less objective and more sensitive to noise.

No correlations were found between the three MRI methods for the superior, anterior position of both lungs. A possible reason for this finding is that this region of the lung, as defined in the methods, is the lowest percentage of the total lung volume and, therefore, the appearance or lack of ventilation defects could disproportionally increase or decrease the estimated VDP values, respectively. Although, in this region of the lung, a higher median VDP_Xe_ was found as compared 3D and 2D VDP_RVent_. Therefore, the inherent differences of the three methods are likely a large contributing factor to the lack of correlation in certain regions, and some spatial discordance between PREFUL and Xe‐MRI has been previously reported, although overall defect burdens are largely comparable. Future studies investigating these different imaging methods will help to understand this variability. Additionally, incorporation of structural imaging may help to determine underlying sources of any potential discordance.

In this study, Xe‐MRI was used as a reference for assessing regional pulmonary ventilation in pediatric CF lung disease to compare to VDP derived from 3D UTE PREFUL MRI. Although Xe‐MRI and PREFUL MRI moderately correlate in pediatric CF lung disease^14^, ^15^, ^16^ and other pulmonary diseases such as COPD,^12^ the techniques differ in their ability to measure lung function. First, Xe‐MRI is acquired during a breath‐hold at a fixed lung volume of approximately FRC + 1/6th of TLC, whereas PREFUL MRI is conducted during free breathing during tidal respiration. Furthermore, Xe‐MRI quantifies the level of gas mixing within the lungs, regardless of the deformation of the lung parenchymal tissue, and detects ventilation abnormalities associated with slow filling or blocked airways. In contrast, PREFUL MRI assesses the overall alteration in lung tissue signal (i.e., density) throughout the respiratory cycle, potentially capturing both ventilation‐related changes and structural and mechanical changes (e.g., compliance) in the lungs. Therefore, the effect of tissue compression because of gravity at the dependent lung (i.e., the posterior lung in a supine subject) can have a larger impact on PREFUL MRI, which is performed during tidal breathing, as compared to Xe‐MRI acquired during a single‐breath. In a previous study, these effects were posited as a possible source of higher inter‐visit variability with PREFUL MRI.^16^ Future studies of 3D PREFUL MRI will include evaluating the repeatability and longitudinal changes of the proposed method in pediatric CF lung disease compared to of 2D PREFUL MRI.

In this study, neither the VDP from Xe‐MRI or PREFUL MRI significantly correlated with FEV_1_/FVC and FEV_1_. This finding contrasts with a previous study on a separate cohort of stable pediatric CF participants in which single slice VDP_RVent_ correlated weakly with FEV_1_ (*R* ^2^ = 0.12, *p* = 0.02), whereas 2D multi‐slice VDP_Xe_ correlated moderately with FEV_1_ (*R* ^2^ = 0.19, *p* = 0.004).^16^ Possible reasons for the lack of correlation include the low sample size and the low variability of disease severity within the CF cohort under investigation, compared to recent studies in similar populations. Particularly, of the 17 patient visits, only three showed a VDP_Xe_ >10% and four showed a 3D VDP_RVent_ >10%, which is attributable to the effect of ETI treatment on these CF patients. Notably, one CF patient reported an FEV_1_ of 120% predicted, whereas 3D VDP_RVent_, 2D VDP_RVent_, and VDP_Xe_ were measured to be 18.5%, 14.7%, and 10.6%, respectively. These findings are in agreement with past work, which showed that MRI may be more sensitive than spirometry to CF lung disease.^6^ However, 3D VDP_RVent_ and VDP_Xe_, but not 2D VDP_RVent_, significantly correlated with LCI, where VDP_Xe_ showed the strongest correlation to LCI as compared to both PREFUL derived VDP measures. In previous studies of pediatric CF lung disease, VDP_Xe_ also showed a stronger correlation to LCI than single‐slice PREFUL MRI.^14^, ^15^ Therefore, the lowered correlation strength of PREFUL‐derived VDP measures with LCI may not be attributed to the incomplete coverage of the lung by PREFUL MRI, but rather by the differences in which the method assesses pulmonary ventilation as described above.

Overall, the 3D UTE PREFUL MRI method feasibly provided a whole lung assessment of pulmonary ventilation. In comparison to Xe‐MRI, the 3D PREFUL MRI does not require any special equipment (e.g., a polarizer or ^129^Xe coil) and the ability to implement these advanced imaging sequences and reconstruction techniques is easier and more affordable at all sites with ^1^H MR scanning capabilities. Additionally, the PREFUL algorithm is performed on MR images acquired during quiescent tidal breathing and does not require a prolonged breath‐hold. Breath‐holds may be difficult with CF children who have severe lung disease, particularly for those undergoing a pulmonary exacerbation where instances of gas exhalation during an acquisition have been shown.^7^ Image quality can suffer from the aliasing of the exhaled ^129^Xe gas outside of the area of the lung and may influence the quantification of ventilation defects at different sections of the lung. Although PREFUL MRI does require several minutes of relaxed tidal breathing, instances of coughing may be detected using the DC signal and the corresponding data can be rejected from reconstruction in order maintain image quality, thereby making this technique less susceptible to motion corruption. In particular, PREFUL MRI may allow for functional lung imaging in fragile populations such as neonates. Conventional PREFUL MRI has shown feasibility in neonates, imaged within their first week of birth,^35^ including in premature neonates with bronchopulmonary dysplasia.^36^ Although the 3D PREFUL MRI method requires more complex sequence design and reconstruction as compared to the 2D method, the improvement in spatial resolution outweighs these disadvantages, most of which, can be easily automated. Additionally, to provide full coverage of the lung, 2D PREFUL would likely require 10 to 15 min depending on the size of the patient. Therefore, the 3D PREFUL technique described in this work may allow for higher‐resolution and faster functional lung imaging than previously described.

Previous studies have shown the feasibility of 3D and UTE PREFUL MRI techniques, but not the combination of the two. A pseudo‐3D, stack‐of‐stars based PREFUL MRI method was proposed by Klimes et al.^17^ to provide whole lung ventilation assessment. In comparison to the proposed 3D UTE method, the stack‐of‐stars method could be performed by reconstructing 20 to 60 respiratory states. The 3D PREFUL MRI method strongly correlated with the 2D multi‐slice method, but showed higher sensitivity to ventilation heterogeneity in adult patients with pulmonary diseases.^17^ Ventilation parameters derived from 3D PREFUL MRI was also shown to be repeatable in healthy volunteers and COPD patients,^18^ correlate well with spirometry, as well as track improvements in lung function in CF patients beginning CFTR‐modulator treatment.^19^ In another work, PREFUL MRI was also shown to be feasible with a 2D radial UTE (0.07 ms) approach, using an in‐plane resolution of 2.8 mm^2^ and slice thickness of 12.5 mm, in a healthy volunteer.^21^ The authors found that the areas of low ventilation were greater in the UTE acquisition as compared to that of the standard GRE sequence, but were in similar spatial locations.

Although the PREFUL MRI technique showed promising findings in this study, there were limitations. First, the spatial resolution of the 2D multi‐slice ^129^Xe images (2 mm × 2 mm × 15 mm) were different from the 3D UTE images (2 × 2 × 2 mm^3^) used for the PREFUL analysis. Xe‐MRI methods that offer isotropic resolution by using non‐Cartesian trajectories (e.g., spiral/radial)^37^, ^38^ could serve as a better comparator for the 3D PREFUL regional ventilation maps. Additionally, a landmark‐based registration was used to match 3D PREFUL MRI and Xe‐MRI defect maps for spatial correlation, which may lead to inaccurate matching of defect regions. Furthermore, image distortion corrections caused by non‐linearity gradient were not implemented, which could potentially improve the UTE MR image quality. Last, perfusion‐weighted 3D PREFUL MRI was not explored in this study because of the elimination of the blood in‐flow signal of the non‐selective hard RF pulse. Three dimensional lung perfusion has shown some feasibility in healthy volunteers using a volume‐selective RF pulse,^39^ but has not yet been implemented with a PREFUL‐based analysis method. Improvements to the 3D PREFUL MRI approach may include increased undersampling of k‐space to reduce scan time,^17^ thereby reducing acquisitions to ˜4 min, which is of particular importance in children or infants who are prone to spontaneous movement. Additionally, to reduce processing time and reader bias, deep learning‐based segmentation method^40^ could be implemented in place of the semi‐automated seeded region growing method used in this study.

## 5 CONCLUSION

Whole lung ventilation assessment using a 3D free breathing UTE MRI acquisition with the PREFUL algorithm is feasible and shows good global agreement with multi‐slice hyperpolarized ^129^Xe MRI in pediatric CF lung disease and conventional 2D PREFUL MRI on a slice‐matched basis. Regional discordance of ventilation defects were noted and may be expected from the inherent differences of the techniques. Overall, PREFUL MRI provides a more accessible approach compared to Xe‐MRI, which requires an inhaled contrast agent and special equipment, for assessing regional lung function in CF lung disease and may be more advantageous for imaging very sick and young children (e.g., neonates).
